# Barriers to Buprenorphine Dispensing by Medicaid-Participating Community Retail Pharmacies

**DOI:** 10.1001/jamahealthforum.2024.1077

**Published:** 2024-05-17

**Authors:** Patricia R. Freeman, Lindsey R. Hammerslag, Katherine A. Ahrens, Michael Sharbaugh, Adam J. Gordon, Anna E. Austin, Julie M. Donohue, Lindsay D. Allen, Andrew J. Barnes, Jeffery C. Talbert

**Affiliations:** 1 Department of Pharmacy Practice and Science, University of Kentucky, Lexington; 2 Institute for Biomedical Informatics, University of Kentucky College of Medicine, Lexington; 3 Muskie School of Public Service, University of Southern Maine, Portland; 4 Department of Health Policy and Management, School of Public Health, University of Pittsburgh, Pittsburgh, Pennsylvania; 5 Department of Medicine, School of Medicine, University of Utah, Salt Lake City; 6 Gillings School of Public Health, University of North Carolina, Chapel Hill; 7 Feinberg School of Medicine, Northwestern University, Chicago, Illinois; 8 School of Medicine Health Behavior and Policy, Virginia Commonwealth University, Richmond; 9 Associate Editor, *JAMA Health Forum*

## Abstract

**Importance:**

Controlled substances have regulatory requirements under the US Federal Controlled Substance Act that must be met before pharmacies can stock and dispense them. However, emerging evidence suggests there are pharmacy-level barriers in access to buprenorphine for treatment for opioid use disorder even among pharmacies that dispense other opioids.

**Objective:**

To estimate the proportion of Medicaid-participating community retail pharmacies that dispense buprenorphine, out of Medicaid-participating community retail pharmacies that dispense other opioids and assess if the proportion dispensing buprenorphine varies by Medicaid patient volume or rural-urban location.

**Design, Setting, and Participants:**

This serial cross-sectional study included Medicaid pharmacy claims (2016-2019) data from 6 states (Kentucky, Maine, North Carolina, Pennsylvania, Virginia, West Virginia) participating in the Medicaid Outcomes Distributed Research Network (MODRN). Community retail pharmacies serving Medicaid-enrolled patients were included, mail-order pharmacies were excluded. Analyses were conducted from September 2022 to August 2023.

**Main Outcomes and Measures:**

The proportion of pharmacies dispensing buprenorphine approved for opioid use disorder among pharmacies dispensing an opioid analgesic or buprenorphine prescription to at least 1 Medicaid enrollee in each state. Pharmacies were categorized by median Medicaid patient volume (by state and year) and rurality (urban vs rural location according to zip code).

**Results:**

In 2016, 72.0% (95% CI, 70.9%-73.0%) of the 7038 pharmacies that dispensed opioids also dispensed buprenorphine to Medicaid enrollees, increasing to 80.4% (95% CI, 79.5%-81.3%) of 7437 pharmacies in 2019. States varied in the percent of pharmacies dispensing buprenorphine in Medicaid (range, 73.8%-96.4%), with significant differences between several states found in 2019 (χ^2^ *P* < .05), when states were most similar in the percent of pharmacies dispensing buprenorphine. A lower percent of pharmacies with Medicaid patient volume below the median dispensed buprenorphine (69.1% vs 91.7% in 2019), compared with pharmacies with above-median patient volume (χ^2^ *P* < .001).

**Conclusions and Relevance:**

In this serial cross-sectional study of Medicaid-participating pharmacies, buprenorphine was not accessible in up to 20% of community retail pharmacies, presenting pharmacy-level barriers to patients with Medicaid seeking buprenorphine treatment. That some pharmacies dispensed opioid analgesics but not buprenorphine suggests that factors other than compliance with the Controlled Substance Act influence pharmacy dispensing decisions.

## Key Points

**Question:**

Is buprenorphine for opioid use disorder accessible in Medicaid-participating pharmacies that dispense other opioids?

**Findings:**

In this serial cross-sectional study in 6 states, from 2016 to 2019, 72.0% to 80.4% of community retail pharmacies that dispensed other opioids also dispensed buprenorphine.

**Meaning:**

Nearly 1 in 5 pharmacies dispensing other opioids to patients with Medicaid did not dispense buprenorphine, which may present pharmacy-level barriers to patients with Medicaid seeking buprenorphine treatment.

## Teaser

This serial cross-sectional study estimates the proportion of Medicaid-participating pharmacies that dispensed buprenorphine among those dispensing any opioid in 6 states and assesses variation by Medicaid patient volume or rural vs urban location.

## Introduction

Increasing access to buprenorphine treatment for opioid use disorder (OUD) is a major focus of US health policy. To date, interventions have focused on reducing prescriber-level barriers, such as expanding the number and type of prescribers^1^ and removing prior authorization policies in commercial insurance and state Medicaid plans.^2^

Emerging evidence from qualitative interviews,^3^ surveys of community pharmacists,^4^ and secret-shopper studies^5,6^ suggests pharmacy-level barriers to buprenorphine access are important. Pharmacy-level barriers to buprenorphine dispensing may be driven by a variety of factors, including fear of regulatory enforcement action and stigma.^7,8^

Medicaid pharmacy programs provide coverage for outpatient prescription drugs, including buprenorphine. Medicaid claims provide a robust data source for the quantitative assessment of pharmacy buprenorphine dispensing. In this serial cross-sectional study, we estimated the proportion of Medicaid-participating pharmacies that dispensed buprenorphine among those dispensing any opioid in 6 states and assessed variation by Medicaid patient volume or rural vs urban location.

## Methods

We followed the Strengthening the Reporting of Observational Studies in Epidemiology (STROBE) reporting guidelines for reporting this observational study. State identifiers were masked for reporting. This research was determined to be exempt by each participating university’s institutional review board, and data analysis was at the pharmacy claim level and not individual patient level.

Medicaid claims data were obtained from 6 states (Kentucky, Maine, North Carolina, Pennsylvania, Virginia, West Virginia) participating in the Medicaid Outcomes Distributed Research Network (MODRN).^9^ Using the MODRN common data model and a standardized analysis plan, states obtained Medicaid outpatient pharmacy claims for medications dispensed between January 1, 2016, and December 31, 2019; analyses were conducted from September 2022 through the end of August 2023. National Provider Identifiers (NPIs) were used to identify pharmacies dispensing at least 1 opioid analgesic (OA) or buprenorphine in each year. OAs were identified by National Drug Code (NDC) numbers using the 2020 Centers for Disease Control (CDC) Opioid NDC and Oral Morphine Milligram Equivalent (MME) Conversion File.^10^ Buprenorphine formulations indicated by the US Food and Drug Administration for OUD treatment were identified using the NDC list (eMethods in Supplement 1).

Pharmacy business addresses and taxonomy codes of community retail pharmacies (3336C0003X) were obtained using a National Plan and Provider Enumeration System Data Dissemination file. The 2010 US Department of Agriculture’s Rural-Urban Commuting Area (RUCA) zip code–level codes were used to classify pharmacies located in urban (RUCA 1-3) vs rural (RUCA 4-10) areas.^11^ Pharmacy patient volume was determined using the median number of unique Medicaid beneficiaries with any dispensed prescriptions for each state and year.

SAS statistical software (version 9.4, SAS Institute) was used to create the standard program distributed to the participating states for analysis and R statistical software (version 4.3.1, R Foundation) was used to visualize data and generate exact binomial 95% CIs. χ^2^ or Fisher exact tests were used to test for variation in the proportion of pharmacies dispensing buprenorphine, of all pharmacies dispensing OA and/or buprenorphine. Comparisons included (1) between years (2016 vs 2019), (2) between states (in 2019), (3) between years within pharmacy volume group or urban vs rural (2016 vs 2019), and (4) between pharmacy volume group or urban vs rural (in 2016 or 2019). Results were considered statistically significant for 2-sided *P* < .05.

A sensitivity analysis was conducted using the physical location of pharmacies instead of NPI numbers in 1 state, where pharmacy mergers in 2018 led to duplicate NPIs for 20% of physical locations. We also conducted a sensitivity analysis examining pharmacies dispensing at least 10 prescriptions, rather than at least 1. Finally, to contextualize our findings we examined trends in pharmacy dispensing of varenicline, a noncontrolled substance used for tobacco cessation that is also used to treat addiction but is subject to fewer regulatory requirements.

## Results

**Figure 1.**
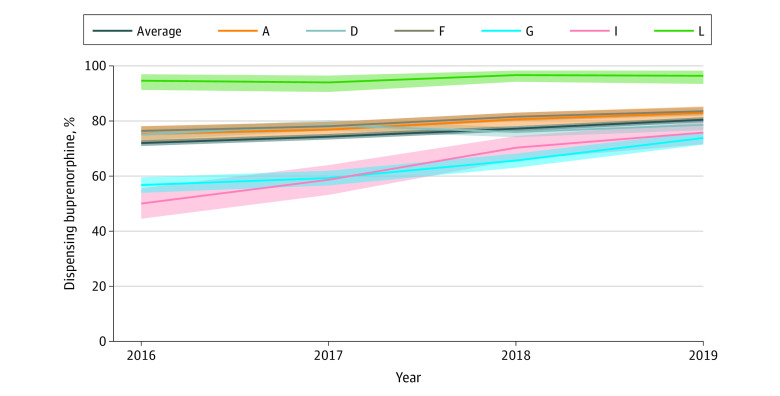
Trends in the Proportion of Pharmacies Dispensing Buprenorphine by State, 2016 to 2019 The proportion of pharmacies dispensing buprenorphine approved for opioid use disorder treatment was calculated using the number of pharmacies dispensing at least 1 opioid analgesic or buprenorphine prescription as the denominator. Any pharmacy National Provider Identifier number that had at least 1 Medicaid claim for a product was considered to have dispensed that product. Shaded areas represent the exact binomial 95% CIs. The letters in the key represent state pseudonyms assigned and reported in prior Medicaid Outcomes Distributed Research Network publications.

The number of pharmacies dispensing OA and/or buprenorphine and the proportion of those pharmacies that dispensed buprenorphine by state and year are shown in the Table. In 2016, 5067 (72.0%) (95% CI, 70.9%-73.0%) of 7038 pharmacies dispensed buprenorphine, increasing to 5979 (80.4%) (95% CI, 79.5%-81.3%) of 7437 in 2019. The proportion dispensing buprenorphine increased in all states across the study period (Figure 1); the increase was significant in all but 1 state (eTable 1 in Supplement 2). States with the lowest proportion dispensing buprenorphine in 2016, states G and I, had large increases in buprenorphine dispensing over time, but both remained significantly lower than states A, F, and L in 2019 (eTable 1 in Supplement 2).

**Table.** Number and Proportion of Pharmacies Dispensing Buprenorphine by State, 2016 to 2019

MODRN state, year	All opioid dispensing pharmacies, No.				Buprenorphine dispensing pharmacies, % (95% CI)^a^			
	2016	2017	2018	2019	2016	2017	2018	2019
State A	861	868	988	978	75.3 (72.2-78.1)	76.8 (73.9-79.6)	80.6 (78.0-83.0)	82.9 (80.4-85.2)
State D	1836	1873	2101	1830	75.1 (73.0-77.0)	78.4 (76.5-80.3)	76.0 (74.1-77.8)	78.6 (76.6-80.4)
State F	2509	2539	2553	2520	76.4 (74.7-78.0)	78.1 (76.4-79.7)	81.5 (80.0-83.0)	83.5 (82.0-85.0)
State G	1222	1330	1364	1371	56.7 (53.9-59.5)	59.2 (56.6-61.9)	65.6 (63.0-68.1)	73.8 (71.4-76.1)
State I	332	336	444	462	50.0 (44.5-55.5)	58.6 (53.2-63.9)	70.3 (65.8-74.5)	75.8 (71.6-79.6)
State L	278	282	358	276	94.6 (91.3-96.9)	94.0 (90.5-96.4)	96.6 (94.2-98.3)	96.4 (93.4-98.2)
Combined total	7038	7228	7808	7437	72.0 (70.9-73.0)	74.3 (73.2-75.3)	77.2 (76.2-78.1)	80.4 (79.5-81.3)

Abbreviation: MODRN, Medicaid Outcomes Distributed Research Network.

^a^ Denominator includes pharmacies dispensing at least 1 opioid analgesic or buprenorphine prescription to at least 1 Medicaid enrollee in that state in that calendar year. Numerator includes pharmacies dispensing at least 1 buprenorphine prescription to at least 1 Medicaid enrollee in that state in that calendar year. CI represents the exact binomial 95% CI.

**Figure 2.**
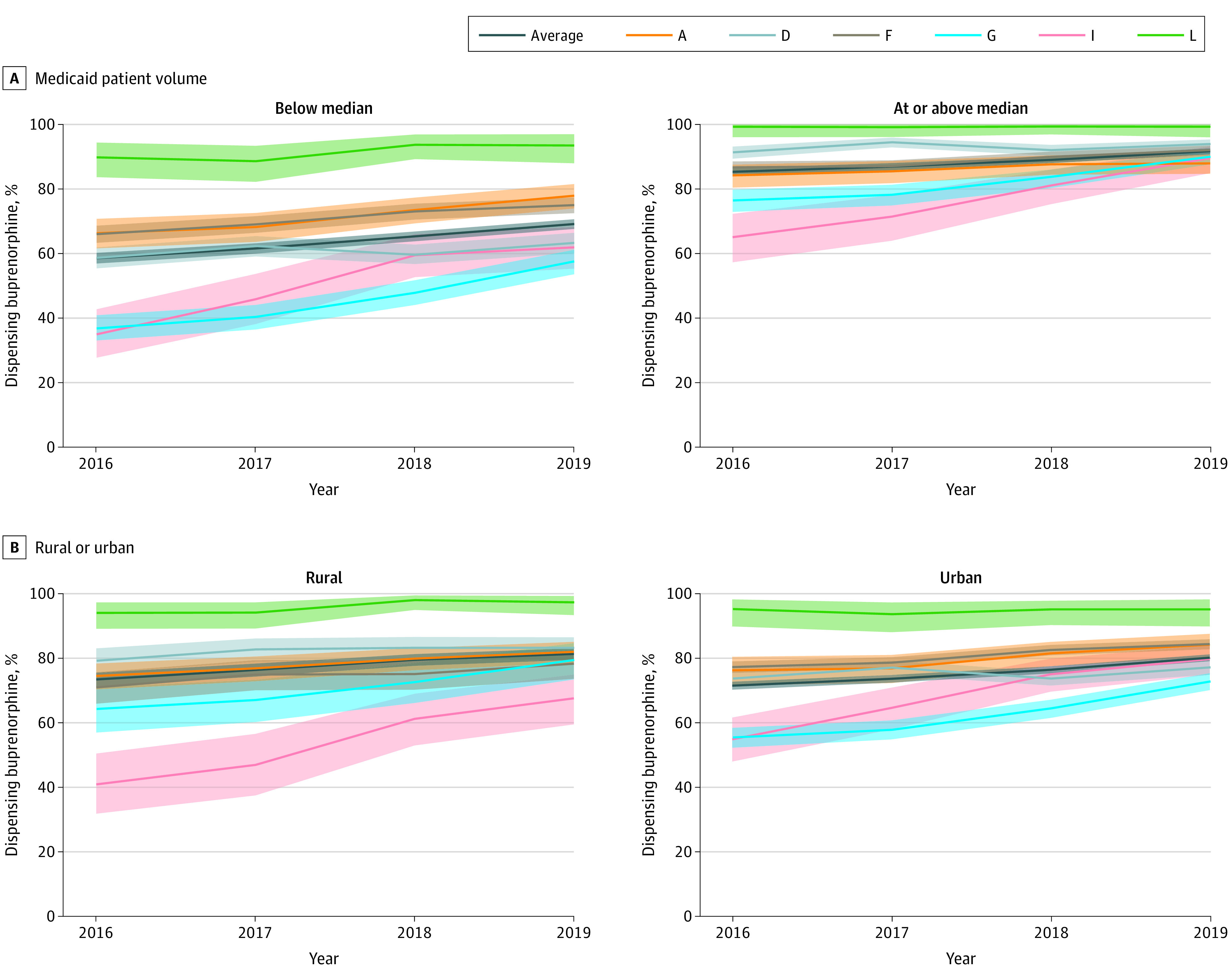
Trends in the Proportion of Pharmacies Dispensing Buprenorphine by State, and Medicaid Patient Volume and Urbanization, 2016 to 2019 Medicaid patient volume was determined using a median split of the total number of unique Medicaid enrollees with dispensed prescriptions associated with each pharmacy National Provider Identifier number, within each state and each year. Urbanization was determined using the zip code for the pharmacy location. Zip codes with primary Rural-Urban Commuting Area (RUCA) codes of 1 to 3 were classified as urban and pharmacies with RUCA codes of 4 to 10 were classified as rural. The proportion of pharmacies dispensing buprenorphine approved for opioid use disorder treatment was calculated using the number of pharmacies dispensing opioid analgesics or buprenorphine as the denominator. Any pharmacy National Provider Identifier number that had at least 1 Medicaid claim for a product was considered to have dispensed that product. Shaded areas represent the exact binomial 95% CIs. The letters in the key represent state pseudonyms assigned and reported in prior Medicaid Outcomes Distributed Research Network publications.

The proportion of pharmacies dispensing buprenorphine varied by Medicaid patient volume (Figure 2A; eTable 2 in Supplement 2). In 2019, 2568 of 3716 pharmacies (69.1%) (95% CI, 67.6%-70.6%) with below median patient volume dispensed buprenorphine compared with 4312 of the 3721 pharmacies (91.7%) (95% CI, 90.8%-92.6%) at or above median volume. From 2016 to 2019, there was a significant increase in the proportion dispensing buprenorphine for both higher and lower volume pharmacies (eTable 3 in Supplement 2), though this varied by state.

The proportion of rural and urban pharmacies dispensing buprenorphine varied by state (Figure 2B; eTable 5 in Supplement 2). Both rural and urban pharmacies had increases in the proportion dispensing buprenorphine over time (eTable 3 in Supplement 2, *P* < .05). Although rurality was not associated with overall dispensing in 2016 or 2019, rural-urban differences reached statistical significance in some states (eTable 4 in Supplement 2).

Results from sensitivity analyses using pharmacy location instead of NPI in 1 state were similar. Trends and state variation using a threshold of 10 instead of 1 prescription fills for buprenorphine-dispensing pharmacies were comparable to the primary analysis but the percent of pharmacies dispensing buprenorphine was lower (eTable 6, eFigures 1-2 in Supplement 2). The percent of pharmacies with any varenicline dispensing in each year was comparable to the percent dispensing buprenorphine (eTable 7, eFigure 3 in Supplement 2).

## Discussion

This is the first study to quantify buprenorphine dispensing in pharmacies using Medicaid claims. Although buprenorphine dispensing increased from 2016 to 2019, only 80.4% of pharmacies that dispensed opioids to Medicaid beneficiaries also dispensed buprenorphine in 2019. Our findings align with previous studies suggesting that buprenorphine may not be fully accessible in up to 30% of pharmacies.^3,4,5,6^ Previous studies suggest pharmacist or pharmacy owner concern over diversion,^8^ fear of regulatory enforcement action by licensure boards and the Drug Enforcement Administration,^12^ and wholesaler thresholds that limit the volume of buprenorphine a pharmacy can order^3,7^ as reasons why pharmacies may not stock and dispense buprenorphine. Results showing that some pharmacies dispensed OAs but not buprenorphine suggest considerations other than the burden of complying with the Controlled Substance Act influence pharmacy dispensing. Recent studies identify pharmacist stigma toward individuals with OUD in treatment with buprenorphine as a barrier to access.^6,13^ However, our finding that rates of pharmacy dispensing for buprenorphine were similar to those of varenicline, a medication used to treat nicotine addiction that is subject to less stigma and regulatory oversight than buprenorphine, suggests that other factors may play an important role.

Although the proportion dispensing buprenorphine increased over time, we found significant variability by state, possibly driven by policy differences. Reimbursement, prior authorization requirements and buprenorphine treatment requirements set by state Medicaid programs may influence buprenorphine prescribing and dispensing.^14^ State prescribing regulations may also influence buprenorphine dispensing, particularly when oversight requirements exceed federal rules and guidelines.^15^

### Limitations

This study does have some limitations. Because we only had access to Medicaid claims, we were unable to observe dispensing patterns with other payment methods.

## Conclusions

In this serial cross-sectional study of Medicaid-participating pharmacies in 6 states, we identified pharmacy-level barriers to patients with Medicaid seeking buprenorphine treatment for OUD, with 1 in 5 pharmacies not dispensing buprenorphine. The influence of state-level buprenorphine prescribing guidelines, Medicaid policies related to buprenorphine coverage and reimbursement, and pharmacist stigma warrant further investigation.

## Notes

Supplement 1. eMethods

Supplement 2. eTable 1. Chi-squared test results comparing the total proportion of pharmacies dispensing buprenorphine in 2019 to either the same state in 2016, or to different states in 2019 eTable 2. Number and Proportion of Pharmacies Dispensing Buprenorphine by State, Classified by Medicaid Patient Volume, 2016-2019 eTable 3. Chi-squared and Fisher’s Exact test results comparing the total proportion of pharmacies dispensing buprenorphine in 2019 versus 2016, stratified by pharmacy Medicaid patient volume or urbanization eTable 4. Chi-squared and Fisher’s Exact test results comparing the total proportion of pharmacies dispensing buprenorphine as a function of pharmacy patient volume or urbanization, in 2016 or 2019 eTable 5. Proportion of Pharmacies Dispensing Buprenorphine in Rural and Urban Areas by State and Year, 2016-2019 eTable 6. Proportion of Pharmacies Dispensing at least 10 Buprenorphine Prescriptions by State, 2016-2019 eTable 7. Proportion of Pharmacies Dispensing Varenicline by State eFigure 1. Trends in the Proportion of Pharmacies Dispensing at Least 10 Buprenorphine Prescriptions by State, 2016-2019 eFigure 2. Trends in the Proportion of Pharmacies Dispensing at Least 10 Buprenorphine Prescriptions by State, and Medicaid Patient Volume (A) and Urbanization (B), 2016-2019 eFigure 3. Trends in the Proportion of Pharmacies Dispensing Varenicline by State, 2016-2019

Supplement 3. Data Sharing Statement
