# Supplementary material for: Barriers to Buprenorphine Dispensing by Medicaid-Participating Community Retail Pharmacies
Source: JAMA Health Forum. 2024 May 17;5(5):e241077. doi: 10.1001/jamahealthforum.2024.1077 (PMC11102015; doi:10.1001/jamahealthforum.2024.1077)
Supplement: Supplement 2. — eTable 1. Chi-squared test results comparing the total proportion of pharmacies dispensing buprenorphine in 2019 to either the same state in 2016, or to different states in 2019 eTable 2. Number and Proportion of Pharmacies Dispensing Buprenorphine by State, Classified by Medicaid Patient Volume, 2016-2019 eTable 3. Chi-squared and Fisher’s Exact test results comparing the total proportion of pharmacies dispensing buprenorphine in 2019 versus 2016, stratified by pharmacy Medicaid patient volume or urbanization eTable 4. Chi-squared and Fisher’s Exact test results comparing the total proportion of pharmacies dispensing buprenorphine as a function of pharmacy patient volume or urbanization, in 2016 or 2019 eTable 5. Proportion of Pharmacies Dispensing Buprenorphine in Rural and Urban Areas by State and Year, 2016-2019 eTable 6. Proportion of Pharmacies Dispensing at least 10 Buprenorphine Prescriptions by State, 2016-2019 eTable 7. Proportion of Pharmacies Dispensing Varenicline by State eFigure 1. Trends in the Proportion of Pharmacies Dispensing at Least 10 Buprenorphine Prescriptions by State, 2016-2019 eFigure 2. Trends in the Proportion of Pharmacies Dispensing at Least 10 Buprenorphine Prescriptions by State, and Medicaid Patient Volume (A) and Urbanization (B), 2016-2019 eFigure 3. Trends in the Proportion of Pharmacies Dispensing Varenicline by State, 2016-2019 [file jamahealthforum-e241077-s002.pdf]

Supplemental Online Content

Freeman PR, Hammerslag LR, Ahrens KA, et al. Barriers to buprenorphine dispensing by Medicaid-participating community retail pharmacies. *JAMA Health Forum*. 2024;5(5):e241077. doi:10.1001/jamahealthforum.2024.1077

eTable 1. Chi-squared test results comparing the total proportion of pharmacies dispensing buprenorphine in 2019 to either the same state in 2016, or to different states in 2019
eTable 2. Number and Proportion of Pharmacies Dispensing Buprenorphine by State, Classified by Medicaid Patient Volume, 2016-2019
eTable 3. Chi-squared and Fisher's Exact test results comparing the total proportion of pharmacies dispensing buprenorphine in 2019 versus 2016, stratified by pharmacy Medicaid patient volume or urbanization
eTable 4. Chi-squared and Fisher's Exact test results comparing the total proportion of pharmacies dispensing buprenorphine as a function of pharmacy patient volume or urbanization, in 2016 or 2019
eTable 5. Proportion of Pharmacies Dispensing Buprenorphine in Rural and Urban Areas by State and Year, 2016-2019.
eTable 6. Proportion of Pharmacies Dispensing at least 10 Buprenorphine Prescriptions by State, 2016-2019
eTable 7. Proportion of Pharmacies Dispensing Varenicline by State
eFigure 1. Trends in the Proportion of Pharmacies Dispensing at Least 10 Buprenorphine Prescriptions by State, 2016-2019
eFigure 2. Trends in the Proportion of Pharmacies Dispensing at Least 10 Buprenorphine Prescriptions by State, and Medicaid Patient Volume (A) and Urbanization (B), 2016-2019
eFigure 3. Trends in the Proportion of Pharmacies Dispensing Varenicline by State, 2016-2019

This supplemental material has been provided by the authors to give readers additional information about their work.

© 2024 Freeman PR, et al. *JAMA Health Forum.*

eTable 1 – Chi-squared test results comparing the total proportion of pharmacies dispensing buprenorphine in 2019 to either the same state in 2016, or to different states in 2019.

MODRN State, Year of interest	Comparison group for chi-squared tests:						
	Same state, 2016	State A, 2019	State D, 2019	State F, 2019	State G, 2019	State I, 2019	State L, 2019
A, 2019	< 0.001		0.006	0.665	< 0.001	0.001	< 0.001
D, 2019	0.011	0.006		< 0.001	0.002	0.191	< 0.001
F, 2019	< 0.001	0.665	< 0.001		< 0.001	< 0.001	< 0.001
G, 2019	< 0.001	< 0.001	0.002	< 0.001		0.409	< 0.001
I, 2019	< 0.001	0.001	0.191	< 0.001	0.409		< 0.001
L, 2019	0.315	< 0.001	< 0.001	< 0.001	< 0.001	< 0.001	
Combined Total, 2019	< 0.001						

Note: Each test is presented twice in the table above.

© 2024 Freeman PR, et al. *JAMA Health Forum.*

eTable 2. Number and Proportion of Pharmacies Dispensing Buprenorphine by State, Classified by Medicaid Patient Volume, 2016-2019

MODRN State, Pharmacy Size	All Opioid Dispensing Pharmacies, N				Buprenorphine Dispensing Pharmacies, % of Total (95% Confidence Interval)			
	2016	2017	2018	2019	2016	2017	2018	2019
Below median								
State A	430	434	494	489	66.3% (61.6 - 70.7%)	68.2% (63.6 - 72.6%)	73.5% (69.4 - 77.3%)	77.9% (74.0 - 81.5%)
State D	917	936	1,049	915	58.7% (55.4 - 61.9%)	62.3% (59.1 - 65.4%)	59.8% (56.7 - 62.8%)	63.3% (60.1 - 66.4%)
State F	1,253	1,269	1,276	1,260	66.0% (63.3 - 68.6%)	69.0% (66.4 - 71.6%)	73.0% (70.5 - 75.5%)	75.0% (72.5 - 77.4%)
State G	610	664	681	683	36.9% (33.0 - 40.9%)	40.2% (36.5 - 44.1%)	47.9% (44.1 - 51.7%)	57.4% (53.6 - 61.1%)
State I	166	168	222	231	34.9% (27.7 - 42.7%)	45.8% (38.1 - 53.7%)	59.5% (52.7 - 66.0%)	61.9% (55.3 - 68.2%)
State L	139	141	179	138	89.9% (83.7 - 94.4%)	88.7% (82.2 - 93.4%)	93.9% (89.3 - 96.9%)	93.5% (88.0 - 97.0%)
**Combined Total**	3,515	3,612	3,901	3,716	58.5% (56.9 - 60.2%)	61.6% (60.0 - 63.2%)	65.3% (63.8 - 66.8%)	69.1% (67.6 - 70.6%)
At or above median								
State A	431	434	494	489	84.2% (80.4 - 87.5%)	85.5% (81.8 - 88.7%)	87.7% (84.4 - 90.4%)	87.9% (84.7 - 90.7%)
State D	919	937	1,052	915	91.4% (89.4 - 93.1%)	94.6% (92.9 - 95.9%)	92.1% (90.3 - 93.7%)	93.9% (92.1 - 95.3%)
State F	1,256	1,270	1,277	1,260	86.7% (84.7 - 88.5%)	87.1% (85.1 - 88.9%)	90.0% (88.2 - 91.6%)	92.1% (90.4 - 93.5%)
State G	612	666	683	688	76.5% (72.9 - 79.8%)	78.2% (74.9 - 81.3%)	83.3% (80.3 - 86.0%)	90.1% (87.6 - 92.2%)
State I	166	168	222	231	65.1% (57.3 - 72.3%)	71.4% (64.0 - 78.1%)	81.1% (75.3 - 86.0%)	89.6% (84.9 - 93.2%)
State L	139	141	179	138	99.3% (96.1 - 100.0%)	99.3% (96.1 - 100.0%)	99.4% (96.9 - 100.0%)	99.3% (96.0 - 100.0%)
**Combined Total**	3,523	3,616	3,907	3,721	85.3% (84.1 - 86.5%)	86.9% (85.8 - 88.0%)	89.0% (88.0 - 90.0%)	91.7% (90.8 - 92.6%)

Caption: Patient volume was determined using a median split of the total number of unique Medicaid enrollees with dispensed prescriptions associated with each pharmacy National Provider Identifier number, within each state and each year. The proportion of pharmacies dispensing buprenorphine approved for opioid use disorder treatment was calculated using the total (number of pharmacies dispensing opioid analgesics or buprenorphine) as the denominator. Any pharmacy National Provider Identifier number that had at least one Medicaid claim for a product was considered to have dispensed that product. Confidence interval represents the exact binomial 95% confidence interval.

© 2024 Freeman PR, et al. *JAMA Health Forum.*

eTable 3 – Chi-squared and Fisher's Exact test results comparing the total proportion of pharmacies dispensing buprenorphine in 2019 versus 2016, stratified by pharmacy Medicaid patient volume or urbanization.

MODRN State	2016 versus 2019 comparison results, within the following strata:			
	Below median volume	At or above median volume	Rural	Urban
A	< 0.001	0.103	0.004	0.004
D	0.043	0.042	0.160	0.032
F	< 0.001	< 0.001	0.025	< 0.001
G	< 0.001	< 0.001	< 0.001	< 0.001
I	< 0.001	< 0.001	< 0.001	< 0.001
L	0.284	1.000*	0.164	1.000
Combined Total	< 0.001	< 0.001	< 0.001	< 0.001

*This value represents the p-value from a Fisher's exact test, as the smallest expected cell count in this test was less than 5.

© 2024 Freeman PR, et al. *JAMA Health Forum.*

eTable 4 – Chi-squared and Fisher's Exact test results comparing the total proportion of pharmacies dispensing buprenorphine as a function of pharmacy patient volume or urbanization, in 2016 or 2019.

MODRN State	Year	Below versus at or above median volume	Rural versus urban
A	2016	< 0.001	0.551
D	2016	< 0.001	0.015
F	2016	< 0.001	0.010
G	2016	< 0.001	0.023
I	2016	< 0.001	0.015
L	2016	< 0.001	0.691
**Combined Total**	2016	< 0.001	0.122
A	2019	< 0.001	0.354
D	2019	< 0.001	0.009
F	2019	< 0.001	0.004
G	2019	< 0.001	0.042
I	2019	< 0.001	0.004
L	2019	0.010	0.356*
**Combined Total**	2019	< 0.001	0.269

*This value represents the p-value from a Fisher's exact test, as the smallest expected cell count in this test was less than 5.

© 2024 Freeman PR, et al. *JAMA Health Forum.*

eTable 5. Proportion of Pharmacies Dispensing Buprenorphine in Rural and Urban Areas by State and Year, 2016 – 2019.

MODRN State, Urbanization	All Opioid Dispensing Pharmacies, N				Buprenorphine Dispensing Pharmacies, % of Total (95% Confidence Interval)			
	2016	2017	2018	2019	2016	2017	2018	2019
Rural								
State A	478	488	556	554	74.5% (70.3 - 78.3%)	76.8% (72.8 - 80.5%)	79.9% (76.3 - 83.1%)	81.9% (78.5 - 85.1%)
State D	446	445	487	437	79.4% (75.3 - 83.0%)	82.7% (78.9 - 86.1%)	83.4% (79.8 - 86.6%)	83.1% (79.2 - 86.5%)
State F	351	351	353	350	70.9% (65.9 - 75.6%)	74.9% (70.1 - 79.4%)	75.1% (70.2 - 79.5%)	78.3% (73.6 - 82.5%)
State G	190	212	212	214	64.2% (57.0 - 71.0%)	67.0% (60.2 - 73.3%)	72.6% (66.1 - 78.5%)	79.4% (73.4 - 84.6%)
State I	115	113	152	151	40.9% (31.8 - 50.4%)	46.9% (37.5 - 56.5%)	61.2% (53.0 - 69.0%)	67.5% (59.5 - 74.9%)
State L	153	154	200	151	94.1% (89.1 - 97.3%)	94.2% (89.2 - 97.3%)	98.0% (95.0 - 99.5%)	97.4% (93.4 - 99.3%)
**Combined Total**	1,733	1,763	1,960	1,857	73.4% (71.3 - 75.5%)	76.3% (74.3 - 78.3%)	79.5% (77.6 - 81.3%)	81.3% (79.5 - 83.1%)
Urban								
State A	383	380	432	424	76.2% (71.7 - 80.4%)	76.8% (72.3 - 81.0%)	81.5% (77.5 - 85.0%)	84.2% (80.4 - 87.5%)
State D	1,390	1,428	1,614	1,393	73.7% (71.3 - 76.0%)	77.1% (74.8 - 79.3%)	73.7% (71.5 - 75.9%)	77.2% (74.9 - 79.4%)
State F	2,157	2,187	2,199	2,169	77.2% (75.4 - 79.0%)	78.6% (76.8 - 80.3%)	82.5% (80.9 - 84.1%)	84.4% (82.8 - 85.9%)
State G	1,032	1,118	1,152	1,157	55.3% (52.2 - 58.4%)	57.8% (54.8 - 60.7%)	64.3% (61.5 - 67.1%)	72.8% (70.1 - 75.3%)
State I	217	223	292	311	54.8% (48.0 - 61.6%)	64.6% (57.9 - 70.8%)	75.0% (69.6 - 79.9%)	79.7% (74.8 - 84.1%)
State L	125	128	158	125	95.2% (89.8 - 98.2%)	93.8% (88.1 - 97.3%)	94.9% (90.3 - 97.8%)	95.2% (89.8 - 98.2%)
**Combined Total**	5,304	5,464	5,847	5,579	71.5% (70.2 - 72.7%)	73.6% (72.4 - 74.8%)	76.4% (75.3 - 77.5%)	80.1% (79.1 - 81.2%)

Caption: Urbanization was determined using the ZIP code for the pharmacy location. Zip codes with primary Rural-Urban Commuting Area (RUCA) codes of 1-3 were classified as urban and pharmacies with RUCA codes of 4-10 were classified as rural. The proportion of pharmacies dispensing buprenorphine approved for opioid use disorder treatment was calculated using the number of pharmacies dispensing opioid analgesics or buprenorphine as the denominator. Any pharmacy National Provider Identifier number that had with at least one Medicaid claim for a product was considered to have dispensed that product. Confidence interval represents the exact binomial 95% confidence interval.

© 2024 Freeman PR, et al. *JAMA Health Forum.*

eTable 6. Proportion of Pharmacies Dispensing at least 10 Buprenorphine Prescriptions by State, 2016 – 2019.

MODRN State, Year	All Opioid Dispensing Pharmacies, N				Buprenorphine Dispensing Pharmacies, % of Total (95% Confidence Interval)			
	2016	2017	2018	2019	2016	2017	2018	2019
State A	851	858	981	966	67.9% (64.7 - 71.0%)	70.6% (67.5 - 73.7%)	74.8% (72.0 - 77.5%)	78.5% (75.7 - 81.0%)
State D	1,797	1,817	1,984	1,762	64.6% (62.3 - 66.8%)	62.5% (60.2 - 64.7%)	60.1% (57.9 - 62.2%)	68.4% (66.2 - 70.6%)
State F	2,475	2,507	2,497	2,455	66.1% (64.2 - 67.9%)	68.8% (67.0 - 70.6%)	72.4% (70.6 - 74.1%)	74.9% (73.1 - 76.6%)
State G	1,165	1,202	1,245	1,294	38.6% (35.8 - 41.5%)	46.3% (43.5 - 49.2%)	52.0% (49.2 - 54.8%)	60.9% (58.2 - 63.6%)
State I	328	331	439	452	38.7% (33.4 - 44.2%)	55.3% (49.8 - 60.7%)	66.1% (61.4 - 70.5%)	72.3% (68.0 - 76.4%)
State L	275	277	352	273	92.0% (88.1 - 94.9%)	93.5% (89.9 - 96.1%)	90.6% (87.1 - 93.5%)	96.3% (93.4 - 98.2%)
**Combined Total**	6,891	6,992	7,498	7,202	61.0% (59.8 - 62.2%)	63.9% (62.7 - 65.0%)	66.5% (65.5 - 67.6%)	71.9% (70.9 - 72.9%)

Caption: Denominator includes pharmacies dispensing at least 10 opioid analgesic and/or buprenorphine prescriptions to at least 1 Medicaid enrollee in that state in that calendar year. Numerator includes pharmacies dispensing at least 10 buprenorphine prescriptions to at least 1 Medicaid enrollee in that state in that calendar year. Confidence interval represents the exact binomial 95% confidence interval.

© 2024 Freeman PR, et al. *JAMA Health Forum.*

eTable 7. Proportion of Pharmacies Dispensing Varenicline by State

MODRN State, Year	All Opioid Dispensing Pharmacies, N				Varenicline Dispensing Pharmacies, % of Total (95% Confidence Interval)			
	**2016**	**2017**	**2018**	**2019**	**2016**	**2017**	**2018**	**2019**
State A	861	868	988	978	92.1% (90.1 - 93.8%)	94.1% (92.3 - 95.6%)	95.3% (93.8 - 96.6%)	95.6% (94.1 - 96.8%)
State D	1,836	1,873	2,101	1,830	74.3% (72.3 - 76.3%)	74.8% (72.8 - 76.8%)	69.5% (67.5 - 71.5%)	74.8% (72.7 - 76.7%)
State F	2,509	2,539	2,553	2,520	77.1% (75.4 - 78.7%)	80.8% (79.2 - 82.3%)	84.0% (82.5 - 85.4%)	83.0% (81.5 - 84.4%)
State G	1,222	1,330	1,364	1,371	44.6% (41.8 - 47.4%)	47.3% (44.6 - 50.0%)	62.6% (60.0 - 65.2%)	78.4% (76.1 - 80.6%)
State I	332	336	444	462	35.8% (30.7 - 41.3%)	19.3% (15.3 - 24.0%)	73.4% (69.1 - 77.5%)	79.7% (75.7 - 83.2%)
State L	278	282	358	276	92.8% (89.1 - 95.6%)	95.0% (91.8 - 97.3%)	92.5% (89.2 - 95.0%)	95.7% (92.5 - 97.7%)
**Combined Total**	7,038	7,228	7,808	7,437	71.2% (70.2 - 72.3%)	72.4% (71.3 - 73.4%)	77.6% (76.7 - 78.5%)	82.0% (81.1 - 82.9%)

Caption: Denominator includes pharmacies dispensing at least 1 opioid analgesic or buprenorphine prescription to at least 1 Medicaid enrollee in that state in that calendar year. Numerator includes pharmacies dispensing at least 1 varenicline prescription to at least 1 Medicaid enrollee in that state in that calendar year, within the pharmacies included in the denominator. Confidence interval represents the exact binomial 95% confidence interval.

© 2024 Freeman PR, et al. *JAMA Health Forum.*

eFigure 1. Trends in the Proportion of Pharmacies Dispensing at Least 10 Buprenorphine Prescriptions by State, 2016-2019

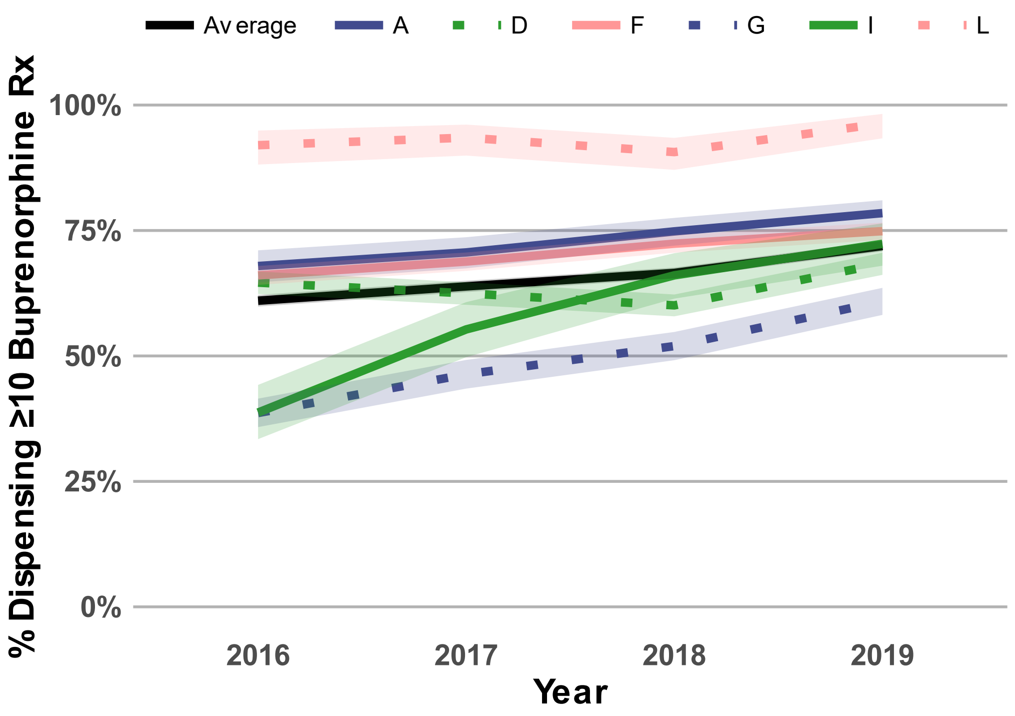

Caption: The proportion of pharmacies dispensing at least 10 prescriptions of buprenorphine approved for opioid use disorder treatment was calculated using the number of pharmacies dispensing at least 10 prescriptions of opioid analgesics or buprenorphine as the denominator. Any pharmacy National Provider Identifier number that had at least 10 Medicaid claims for a product was considered to have dispensed that product. Error bars represent the exact binomial 95% confidence interval.

© 2024 Freeman PR, et al. *JAMA Health Forum.*

eFigure 2. Trends in the Proportion of Pharmacies Dispensing at Least 10 Buprenorphine Prescriptions by State, and Medicaid Patient Volume (A) and Urbanization (B), 2016-2019.

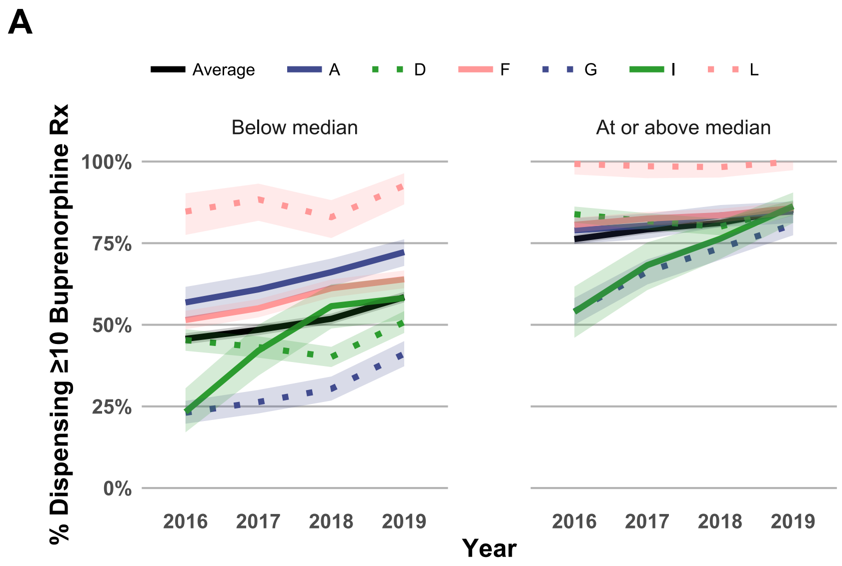

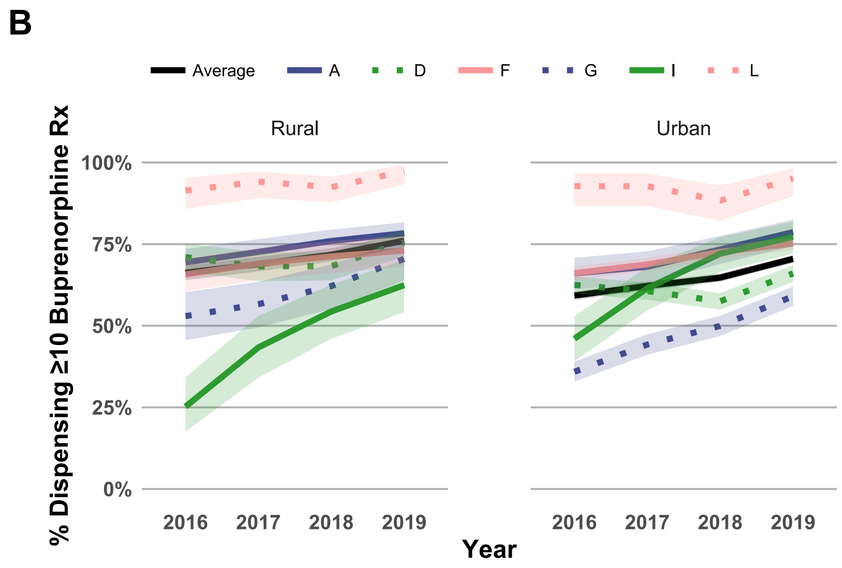

Caption: Medicaid patient volume was determined using a median split of the total number of unique Medicaid enrollees with dispensed prescriptions associated with each pharmacy National Provider Identifier number, within each state and each year. Urbanization was determined using the ZIP code for the pharmacy location. Zip codes with primary Rural-Urban Commuting Area (RUCA) codes of 1-3 were classified as urban and pharmacies with RUCA codes of 4-10 were classified as rural. The proportion of pharmacies dispensing at least 10 prescriptions for buprenorphine approved for opioid use disorder treatment was calculated using the number of pharmacies dispensing at least 10 prescriptions for opioid analgesics or buprenorphine as the denominator. Any pharmacy National Provider Identifier number that had at least 10 Medicaid claims for a product was considered to have dispensed that product. Error bars represent the exact binomial 95% confidence interval.

© 2024 Freeman PR, et al. *JAMA Health Forum.*

eFigure 3. Trends in the Proportion of Pharmacies Dispensing Varenicline by State, 2016-2019.

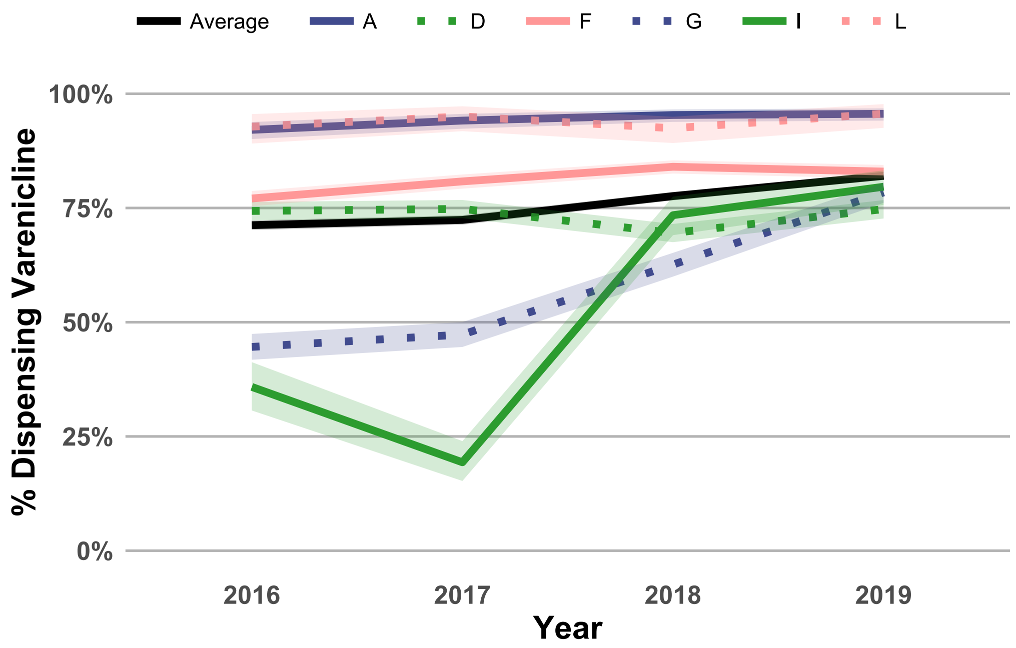

Caption: The proportion of pharmacies dispensing at least one prescription for varenicline approved for tobacco cessation was calculated using the number of pharmacies dispensing opioid analgesics or buprenorphine as the denominator. Error bars represent the exact binomial 95% confidence interval.

© 2024 Freeman PR, et al. *JAMA Health Forum.*
