# Supplementary material for: Barriers to Buprenorphine Dispensing by Medicaid-Participating Community Retail Pharmacies
Source: JAMA Health Forum. 2024 May 17;5(5):e241077. doi: 10.1001/jamahealthforum.2024.1077 (PMC11102015; doi:10.1001/jamahealthforum.2024.1077)
Supplement: Supplement 3. — Data Sharing Statement [file jamahealthforum-e241077-s003.pdf]

Data Sharing Statement

Freeman. Barriers to Buprenorphine Dispensing by Medicaid-Participating Community Retail Pharmacies. *JAMA Health Forum*. Published May 17, 2024.
doi:10.1001/jamahealthforum.2024.1077

Data
Data available: No

Additional Information
Explanation for why data not available: Sharing of data not authorized per state data use agreements.
